# ECDC Round Table Report and ProMed-mail most useful international information sources for the Netherlands Early Warning Committee

**DOI:** 10.2807/1560-7917.ES.2017.22.14.30502

**Published:** 2017-04-06

**Authors:** Paul Bijkerk, Annelie A Monnier, Ewout B Fanoy, Katina Kardamanidis, Ingrid HM Friesema, Mirjam J Knol

**Affiliations:** 1 National Institute for Public Health and the Environment, Bilthoven, The Netherlands; 2 VU University, Amsterdam, The Netherlands; 3 Public Health Service, GGD Region Utrecht, Zeist, The Netherlands

**Keywords:** Europe, The Netherlands, surveillance, epidemiology, early warning

## Abstract

The Netherlands Early Warning Committee (NEWC) aims to identify infectious diseases causing a potential threat to Dutch public health. Threats are assessed and published as (information) alerts for public health experts. To identify threats from abroad, the NEWC screens 10 sources reporting disease outbreaks each week. To identify the sources essential for complete and timely reporting, we retrospectively analysed 178 international alerts published between 31 January 2013 and 30 January 2014. In addition, we asked the four NEWC coordinators about the required time to scan the information sources. We documented the date and source in which the signal was detected. The ECDC Round Table (RT) Report and ProMED-mail were the most complete and timely sources, reporting 140 of 178 (79%) and 121 of 178 (68%) threats respectively. The combination of both sources reported 169 (95%) of all threats in a timely manner. Adding any of the other sources resulted in minor increases in the total threats found, but considerable additional time investment per additional threat. Only three potential relevant threats (2%) would have been missed by only using the ECDC RT Report and ProMed-mail. We concluded that using only the ECDC RT Report and ProMed-mail to identify threats from abroad maintains a sensitive Early Warning System.

## Introduction

Infectious disease outbreaks are threats to public health that usually come unexpectedly and can have considerable consequences especially in case of epidemics and/or pandemics [1]. The Netherlands Early Warning Committee (NEWC) was established in 1999 at the National Institute for Public Health and the Environment (RIVM), in order to identify threats to public health caused by infectious diseases in the Netherlands, in a timely and complete fashion [2]. The weekly NEWC report aims to inform health professionals in order to improve infectious disease prevention and control in the Netherlands through enhancing awareness and ensuring the early detection and reporting of new cases or events.

The NEWC was evaluated in 2006 and 2008 [2,3]. In 2006, a retrospective and descriptive evaluation was performed on the completeness of threat detection in the Netherlands by the NEWC. It was found that the NEWC recognised nearly all national threats in a complete and timely manner. In addition, in 2008, a retrospective descriptive study was performed on the value of ProMed-mail for the NEWC. It was concluded that ProMED-mail has an added value over other sources used by the NEWC in the early detection of threats. Furthermore, ProMED-mail was appreciated for providing background and preliminary outbreak information.

The coordinator of the NEWC scans 10 international sources once a week and selects infectious disease threats based on criteria outlined in a NEWC protocol (available from the authors on request). These criteria are: (i) an unexpected change in the incidence or prevalence of infectious disease; (ii) the occurrence of an infectious disease within a specific population or in a specific location; (iii) the emergence of a new or unknown disease; (iv) an unexpected change in the prevention, treatment or diagnosis of an infectious disease; (v) expected problems or obstacles in the prevention and control of the disease; (vi) an infectious disease threat receiving attention in the media.

During weekly meetings, the NEWC assesses the gathered information from the 10 international information sources (Table 1), decides whether the event is a direct or potential threat to Dutch public health and determines if additional information is needed or whether prevention or control measures need to be taken [4]. The weekly meeting of the NEWC takes place at the National Institute for Public Health and the Environment (RIVM). The participants are microbiologists, epidemiologists and consultants in communicable disease control from various RIVM departments, as well as representatives from the Dutch Food Safety Authority.

**Table 1:** International information sources used by the Netherlands Early Warning Committee, January 2013–January 2014

Organisation	Bulletin / report	Website	Frequency
World Health Organization	Weekly Epidemiological Records (WER)	http://www.who.int/wer/en/	Weekly
	Disease Outbreak News (DON)	http://www.who.int/csr/don/en/	Not applicable^a^
	Event Information Site for International Health Regulations (EIS)	http://apps.who.int/ihr/eventinformation/?ReturnHomeURL=./IHR/CurrentEvents.aspx	Not applicable^a^
European Union or European Centre for Disease Prevention and Control (ECDC)	ECDC Round Table Report	Controlled circulation by Email	Workdays
	*Eurosurveillance*	http://www.eurosurveillance.org/	Weekly
	European Early Warning and Response System (EWRS)^b^	https://ewrs.ecdc.europa.eu/Default.aspx	Not applicable^a^
	Epidemic Intelligence Information System for Food- and Waterborne Diseases and Zoonoses (EPIS FWD)	http://zwpepishome.ecdcdmz.europa.eu/fwd	Not applicable^a^
United States Centers for Disease Control and Prevention (US CDC)	Morbidity and Mortality Weekly Report (MMWR)	http://www.cdc.gov/mmwr/	Weekly
International Society for Infectious Diseases (ISID)	ProMED-mail	http://www.promedmail.org/	Not applicable^a^
Public Health England (PHE)	Emerging Infection (EI) Summary	Controlled circulation by Email	Monthly

^a^ Not applicable: appears only when there is an infectious disease threat or an update from it.

^b^ Operated by ECDC on behalf of the European Commission.

The Dutch weekly electronic reports ‘Wekelijks overzicht van Infectieziektesignalen’ (Weekly overview of infectious diseases signals) are sent by email to ca 2,300 professionals working in the field of infectious diseases in the Netherlands [2]. They are confidential and their access is restricted to infectious disease professionals. Four coordinators of the NEWC rotate weekly in preparing, chairing and writing the report. In this study, we evaluate the usefulness, in terms of completeness and timeliness, and the time required to screen all 10 international information sources by the NEWC.

## Methods

All potential international threats to Dutch public health from abroad reported in the NEWC report between 31 January 2013 and 30 January 2014 were retrospectively analysed. During this 1-year period, the NEWC published 160 international threats. For each published threat, we determined in which of the international information sources listed in Table 1 the threat was described, and at which date the threat was published in both the source and the NEWC report. For each information source, the date of the first description of the threat with the same/closest possible number of cases in that specific geographic area was used in the analysis.

Several threats were subdivided because a pathogen caused outbreaks in different countries or several pathogens caused outbreaks in one country, leading to 47 additional threats for the analysis. We excluded 29 threats either because they (i) were not mentioned in one of the ten sources screened (n = 12); (ii) described an outbreak that took place before the study period (n = 6); (iii) described a policy change concerning a specific disease (n = 1); (iv) were a follow-up of a threat reported in a period before the study period without new cases (n = 6); (v) were about a Dutch patient linked to an international outbreak (n = 2); or (vi) were not correctly archived in our database (n = 2). The 12 threats which were not mentioned in one of the 10 sources screened were found through, for example, expert networks of RIVM experts. This led to a total of 178 threats included in the analysis.

### Definitions

Complete reporting was defined as the number of threats that were reported in each of the 10 information sources. Completeness for each of the sources was the fraction of events covered over total events. Timeliness of reporting was based on whether the publication date of the threat in the information source was before the publication date of the threat in the NEWC report. Furthermore, we asked the four coordinators of the NEWC about the time required to scan the 10 information sources.

### Analyses performed

We performed descriptive analyses and calculated overlap between sources. We analysed in a cumulative way how many additional threats were found when adding an information source, and related this to the time spent for scanning the respective sources. Finally, we evaluated the relevance of missed threats when only scanning a limited number of information sources. Relevance for the Netherlands of missed threats was evaluated based on criteria outlined in the NEWC protocol.

## Results

The percentage of NEWC threats reported in the 10 international information sources used by the NEWC and time interval in days between report in information source and NEWC publication are shown in Table 2.

**Table 2:** Percentage of NEWC threats reported in the 10 international information sources used by the NEWC (n = 178) and time interval in days between report in information source and NEWC publication, the Netherlands, January 2013–January 2014

Information source	Threats reported before NEWC publication			Threats reported after NEWC publication			Reported		Not reported n (%)	
	**N**	**Percentage (%)**	**Time interval in days, median (min-max)**	**N**	**Percentage (%)**	**Time interval in days, median (min-max)**	**N**	**Percentage (%)**	**N**	**Percentage (%)**
ECDC Round Table Reports	140	79	3 (0-129)	4	2	5 (4-53)	144	81	34	19
ProMED-mail	121	68	3 (0-130)	11	6	7 (1-31)	132	74	46	26
WHO Event Information Site (EIS)	45	25	3 (0-361)	12	7	7 (1-61)	57	32	121	68
EPIS for Food- and Waterborne Diseases and Zoonoses (EPIS-FWD)	35	20	7 (0-195)	6	3	13.5 (1-65)	41	23	137	77
WHO Disease Outbreak News (DON)	34	19	3 (0-19)	3	2	5 (1-11)	37	21	141	79
European Early Warning and Response System (EWRS)	32	18	4 (0-367)	7	4	11 (1-160)	39	22	139	78
Eurosurveillance	13	7	7 (7-21)	23	13	49 (7-231)	36	20	142	80
Emerging Infections (EI) Summary	11	6	6 (1-97)	66	37	17 (1-85)	77	43	101	57
Morbidity and Mortality Weekly Report (MMWR)	2	1	9.5 (6-13)	9	5	25 (4-127)	11	6	167	94
WHO Weekly Epidemiological Records (WER)	1	1	NC	4	2	106 (8-204)	5	3	173	97

ECDC: European Centre for Disease Prevention and Control; EPIS: Epidemic Intelligence Information System; NC: not calculable; NEWC: The Netherlands Early Warning Committee; WHO: World Health Organization.

The three international information sources with the highest percentage of complete and timely reporting were the ECDC RT Report (79%), ProMED-mail (68%) and the WHO Event Information Site (25%). Low percentages of complete and timely reporting were found for the WHO Weekly Epidemiological Records (0.6%) and the United States Centers for Disease Control and Prevention (US CDC) Morbidity and Mortality Weekly Report (MMWR) (1%). When only looking at completeness of reporting, the ECDC RT Report (81%), ProMED-mail (74%) and United Kingdom (UK) Emerging Infection (EI) Summary (43%) scored best.

Table 3 shows the average time spent by the coordinators for scanning the information sources. The total time spent on a weekly basis was 230 min. The time spent was least for the WHO Epidemiological Record and the CDC Morbidity and Mortality Weekly Record with both an average of 10 min per week. Most time consuming to scan were the ECDC RT Report, ProMed-mail and the European Early Warning and Response System (EWRS), with an average of 40, 35 and 30 min per week respectively.

**Table 3:** Range of required time for screening 10 international information sources screened by the coordinators of the NEWC (n = 4) in minutes per week, the Netherlands, January 2013– January 2014

International source	Range of time requirement in minutes	Average time requirement in minutes
ECDC Round Table Report	< 15–60	40
ProMED-mail	< 15–45	35
WHO Event Information Site (EIS)	< 15–30	25
EPIS for Food- and Waterborne Diseases and Zoonoses (EPIS-FWD)	< 15–30	15
WHO Disease Outbreak News (DON)	< 15–30	20
European Early Warning and Response System (EWRS)	< 15–40	30
*Eurosurveillance*	< 15–30	25
Emerging Infections (EI) Summary	< 15–45	20
Morbidity and Mortality Weekly Report (MMWR)	< 15	10
WHO Weekly Epidemiological Records (WER)	< 15	10
**TOTAL**	**150–330**	**230**

ECDC: European Centre for Disease Prevention and Control; EPIS: Epidemic Intelligence Information System; NEWC: The Netherlands Early Warning Committee; WHO: World Health Organization.

In the Figure we present the cumulative percentage of timely reported threats in the 10 different international information sources and the average required time per week to scan these sources.

**Figure:**
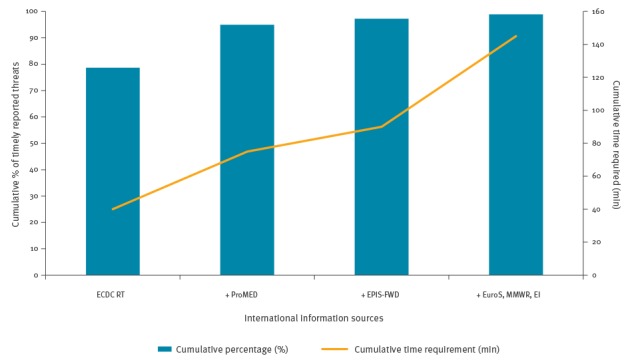
Cumulative percentage of timely reported threats in 10 international information sources screened by the coordinators of the NEWC and time required starting with ECDC Round Table Report and adding different sources, the Netherlands, January 2013– January 2014

ECDC RT: European Centre for Disease Prevention and Control Round Table Report; EPIS-FWD: Epidemic Intelligence Information System for Food- and Waterborne Diseases and Zoonoses; EuroS: *Eurosurveillance*; MMWR: Mortality and Morbidity Weekly Report; EI: Emerging Infections Summary; NEWC: The Netherlands Early Warning Committee.

The Figure shows that scanning the ECDC RT Report only, yielded 140 timely reported threats (79%), with 40 min per week spent on the scanning process. By also scanning ProMED-mail, the NEWC would have detected another 29 timely reported threats, a cumulative percentage of 95% (n = 169 threats), adding another 35 min to the scanning process. By also adding Epidemic Intelligence Information System for Food- and Waterborne Diseases and Zoonosis (EPIS-FWD), four additional timely reported threats would have been detected, adding up to a total of 173 threats (97%), with 15 min of additional time per week. Adding *Eurosurveillance*, the MMWR and the EI Summary would have only yielded three additional timely reported threats, with 55 min in total of additional scanning time per week. Using the ECDC RT Report and ProMED-mail as the sole two international information sources, we would have missed or missed in a timely matter nine threats that would have been detected later, but this would have saved 165 min (72% of the scanning time) per week.

Of the nine threats that we would have missed or missed in a timely matter if we only screened the ECDC RT Report and ProMED-mail, three threats were considered relevant for the Netherlands.

The first was a dengue outbreak involving ca 112 cases (of which 31 confirmed) on the Island of Saint Martin that started in the beginning of January 2013. This outbreak was picked up by the NEWC through their expert network (personal communication, Hans van den Kerkhof, January 2013). The ECDC RT Report of 31 January 2013 mentioned an ‘ongoing outbreak’ on the island. This outbreak was considered relevant because of Dutch travellers to the Dutch Caribbean Islands.

The second reported the detection of wild poliovirus type 1 (WPV 1) in sewerage water in Israel in June 2013 [5]. This threat was reported by WHO Disease Outbreak News [6]. Polio is relevant for the Netherlands because of an existing cluster of unvaccinated people who oppose vaccination for religious reasons, in a certain Dutch region [7]. The ECDC RT Report of 5 September 2013 reported two detections of WPV 1 in April and in August 2013, respectively.

The third threat was about the detection of Seoul Hantavirus in pet rats in Wales (UK). This detection was first described in *Eurosurveillance* [8]. This threat was considered relevant because it was unknown whether these rats were imported to the Netherlands.

The other six threats that we would have missed or missed in a timely matter were not considered relevant for the Netherlands because these threats were local issues within a single European country.

By only screening ECDC RT Report and ProMED-mail, three threats would have been detected with delay. Two of these were first reported in EPIS-FWD, and featured in ECDC RT Report four days after the NEWC report. So when only screening ECDC RT Report and ProMED-mail, these two threats would have been reported one week later in the next NEWC report. One concerned an outbreak of hepatitis A that started in Denmark and was caused by contaminated, frozen berries. These berries were distributed to Sweden where hepatitis A cases were also notified [9]. One other threat that was neither reported in time by the ECDC RT Report or ProMED-mail, nor by any of the other sources. It was picked up by the NEWC through their expert network. The threat in question was a norovirus outbreak in Denmark caused by frozen raspberries. These raspberries were grown in Serbia, packed in Poland and distributed to other northern European countries (personal communication, Harry Vennema, January 2014). No cases were found in the Netherlands.

## Discussion

Our study showed that the Daily ECDC RT Report and ProMED-mail were the most complete and timely sources to identify infectious disease threats from abroad. The combination of both sources resulted in 169 (95%) timely reported threats with only six missed threats and three threats not detected in a timely manner. We found that screening of all 10 sources takes 230 min per week, compared with 65 min per week when we would only use the ECDC Round Table Reports and ProMed-mail.

For the Netherlands, we showed that in order to detect international threats for our weekly report, it is enough to only screen the ECDC Round Table Report and ProMED-mail. That does not mean that the other sources are not valuable with regard to communicating infectious disease threats. Other sources have other strengths, assets or have other aims, such as *Eurosurveillance,* which is a scientific journal with a wide audience. EWRS is a confidential system which allows European Union and European Economic Area (EU/EEA) countries to send alerts about threats with a potential impact on the EU/EEA and to share information between countries. This is also the case for the WHO Event Information Site, where countries have to report public health events under the International Health Regulations [10]. For early warning and response activities, scanning on a daily basis of EWRS and WHO-EIS is useful. In addition, other sources can provide more details about specific threats.  An advantage of sources contributing only very few additional threats may be the timeliness by which they provide a signal, which may be picked up by other sources somewhat later. We found that by exclusively using the ECDC RT Report and ProMED-mail, only three threats were not detected in a timely manner. These three threats were detected 4–7 days later in one of these two sources.

Internationally, to our best knowledge, evaluation studies on sources of Early Warning Systems have not been performed. There are some published studies on the development of Internet surveillance systems for the early identification of health threats (‘epidemic intelligence’) [11-17].

The Early Warning process for the EU is managed by the European Centre for Disease Prevention and Control (ECDC) on behalf of the European Commission. ECDC was established to help strengthen Europe’s defences against infectious diseases, with surveillance and keeping track of health threats inside and outside Europe as one of its core tasks. The Centre is tracking threats through epidemic intelligence. It is screening official and unofficial sources on a 24/7 basis. The Daily RT meeting is the key organisational mechanism in ECDC for initial assessment of acute health threats. The Daily RT has a restricted access; a confidential report is distributed to the nominated Member States’ competent bodies for threat detection, preparedness and response, the World Health Organization, and some national centres for disease control. In addition, since 2012, ECDC has published a weekly publicly available CDTR (Communicable Disease Threats Report) on its website providing updates on threats monitored by ECDC. This weekly report is a summary of the Daily RT reports [18]. The sources which are used by ECDC to produce the Daily RT Report overlap 100% with the sources we use for our NEWC weekly report. ECDC has 10 filtering criteria. One of the main criteria is that an outbreak or event related to communicable diseases extends to more than one EU/EEA country. We have shown that the ECDC RT Report covers almost all international infectious disease threats relevant for the Netherlands. This means that in time of scarce resources at the national level, European countries may consider to rely on the ECDC Daily RT for detecting threats relevant to Europe and its citizens. Consequently, resources at national levels could be shifted to other activities, although this should be assessed by each country individually.

For the first time, an evaluation of international information sources for the NEWC process was performed. We performed a retrospective analysis of the threats and asked the four chairpersons about the time required to scan the 10 information sources. The systematic approach, including the exclusion of e.g. NEWC infectious threats describing only trends and the division of NEWC threats into pathogen- and geographic location-specific threats, ensured high reproducibility of the results.

However, our study has some limitations. Our analysis did not take into account the use of other information sources than the 10 sources on the official list of NEWC sources. For the analysis, it was assumed that a publication date before the publication of the NEWC weekly reports corresponded to the actual use of the information source. This was, however, not necessarily the case. Indeed, timeliness refers to the relative timeliness of the NEWC publication date but not to the date of the event or first report of the event. Access to the ECDC Daily RT Report is restricted. It is not clear if our results can be extrapolated to other European countries, because criteria to select a threat probably differ by country.

Irrespective of the limitations, we conclude that using the ECDC Daily RT Report and ProMed-mail to identify infectious disease threats from abroad allows to maintain complete reporting, only missing three threats which were considered relevant to the Netherlands and would save at least 2.5 hours a week on human resources.

## References

[1] Morens DM Fauci AS Emerging infectious diseases: threats to human health and global stability. PLoS Pathog. 2013;9(7):e1003467. 10.1371/journal.ppat.1003467 23853589 PMC3701702

[2] Rahamat-Langendoen JC van Vliet JA Suijkerbuijk AW Recognition of threats caused by infectious diseases in the Netherlands: the early warning committee. Euro Surveill. 2006;11(12):242-5. 17370963

[3] Zeldenrust ME Rahamat-Langendoen JC Postma MJ van Vliet JA The value of ProMED-mail for the Early Warning Committee in the Netherlands: more specific approach recommended. Euro Surveill. 2008;13(6):74-7. 18445424

[4] Marvin HJ Kleter GA Prandini A Dekkers S Bolton DJ Early identification systems for emerging foodborne hazards. Food Chem Toxicol. 2009;47(5):915-26. 10.1016/j.fct.2007.12.021 18272277

[5] Anis E Kopel E Singer SR Kaliner E Moerman L Moran-Gilad J Insidious reintroduction of wild poliovirus into Israel, 2013. Euro Surveill. 2013;18(38):20586. 10.2807/1560-7917.ES2013.18.38.20586 24084337

[6] World Health Organization (WHO). Poliovirus detected from environmental samples in Israel. Geneva: WHO. [Accessed 4 Jun 2014]. Available from: http://www.who.int/csr/don/2013_06_03/en/

[7] van Wijngaarden JK van Loon AM The polio epidemic in The Netherlands, 1992/1993. Public Health Rev. 1993-1994-1994;21(1-2):107-16. 8041875

[8] Jameson LJ Taori SK Atkinson B Levick P Featherstone CA van der Burgt G Pet rats as a source of hantavirus in England and Wales, 2013. Euro Surveill. 2013;18(9):20415. 23470018

[9] Gillesberg Lassen S Soborg B Midgley SE Steens A Vold L Stene-Johansen K Ongoing multi-strain food-borne hepatitis A outbreak with frozen berries as suspected vehicle: four Nordic countries affected, October 2012 to April 2013. Euro Surveill. 2013;18(17):20467. 23647625

[10] World Health Organization (WHO). International Health Regulations (2005). Third Edition. ISBN: 978 92 4 158049 6. Available from: http://apps.who.int/iris/bitstream/10665/246107/1/9789241580496-eng.pdf

[11] Kaiser R Coulombier D Different approaches to gathering epidemic intelligence in Europe. Euro Surveill. 2006;11(4):E060427.1. 16809830 10.2807/esw.11.17.02948-en

[12] Rotureau B Barboza P Tarantola A Paquet C International epidemic intelligence at the Institut de Veille Sanitaire, France. Emerg Infect Dis. 2007;13(10):1590-2. 10.3201/eid1310.070522 18258016 PMC2851537

[13] Collier N Doan S Kawazoe A Goodwin RM Conway M Tateno Y BioCaster: detecting public health rumors with a Web-based text mining system. Bioinformatics. 2008;24(24):2940-1. 10.1093/bioinformatics/btn534 18922806 PMC2639299

[14] Bohigas PA Santos-O’Connor F Coulombier D Epidemic intelligence and travel-related diseases: ECDC experience and further developments. Clin Microbiol Infect. 2009;15(8):734-9. 10.1111/j.1469-0691.2009.02875.x 19486073

[15] Dente MG Fabiani M Gnesotto R Putoto G Montagna C Simon-Soria F EpiSouth: a network for communicable disease control in the Mediterranean region and the Balkans. Euro Surveill. 2009;14(5):13-6. 19215714 10.2807/ese.14.05.19113-en

[16] Keller M Blench M Tolentino H Freifeld CC Mandl KD Mawudeku A Use of unstructured event-based reports for global infectious disease surveillance. Emerg Infect Dis. 2009;15(5):689-95. 10.3201/eid1505.081114 19402953 PMC2687026

[17] Linge JP Steinberger R Weber TP Yangarber R van der Goot E Al Khudhairy DH Internet surveillance systems for early alerting of health threats. Euro Surveill. 2009;14(13):19162. 19341610

[18] European Centre for Disease Prevention and Control (ECDC). Communicable Disease Threats Report. Stockholm: ECDC. [Accessed 30 Mar 2017]. Available from: http://ecdc.europa.eu/en/publications/surveillance_reports/Communicable-Disease-Threats-Report/Pages/cdtr.aspx

